# Pattern Recognition Software and Techniques for Biological Image Analysis

**DOI:** 10.1371/journal.pcbi.1000974

**Published:** 2010-11-24

**Authors:** Lior Shamir, John D. Delaney, Nikita Orlov, D. Mark Eckley, Ilya G. Goldberg

**Affiliations:** Laboratory of Genetics, National Institute on Aging/National Institutes of Health, Baltimore, Maryland, United States of America; Whitehead Institute, United States of America

## Abstract

The increasing prevalence of automated image acquisition systems is enabling new types of microscopy experiments that generate large image datasets. However, there is a perceived lack of robust image analysis systems required to process these diverse datasets. Most automated image analysis systems are tailored for specific types of microscopy, contrast methods, probes, and even cell types. This imposes significant constraints on experimental design, limiting their application to the narrow set of imaging methods for which they were designed. One of the approaches to address these limitations is pattern recognition, which was originally developed for remote sensing, and is increasingly being applied to the biology domain. This approach relies on training a computer to recognize patterns in images rather than developing algorithms or tuning parameters for specific image processing tasks. The generality of this approach promises to enable data mining in extensive image repositories, and provide objective and quantitative imaging assays for routine use. Here, we provide a brief overview of the technologies behind pattern recognition and its use in computer vision for biological and biomedical imaging. We list available software tools that can be used by biologists and suggest practical experimental considerations to make the best use of pattern recognition techniques for imaging assays.

## Introduction

Computer-aided analysis of microscopy images has been attracting considerable attention in the past few years, particularly in the context of high-content screening (HCS). The link between images and physiology is well established, and it is common knowledge that a significant portion of what we know about biology relies on different types of microscopy and other imaging devices. Automated image acquisition systems integrated with laboratory automation have produced image datasets that are too large for manual processing. This trend led to a new type of biological experiment, in which the image analysis must be performed by machines. Clearly, this approach is different than the bulk of the microscopy performed for the past ∼400 years. However, while the availability of automated microscopy, laboratory automation, computing resources, and digital imaging and storage devices has been increasing consistently, in some cases the bottleneck for high-throughput imaging experiments is the efficacy of computer vision, image analysis, and pattern recognition methods [1]. Computer-based image analysis provides an objective method of scoring visual content independently of subjective manual interpretation, while potentially being more sensitive, more consistent, and more accurate [2]. These advantages are not limited to massive image datasets, as they allow microscopy to be used as a routine assay system even on a small scale.

An effective computational approach to objectively analyze image datasets is pattern recognition (PR, see Box 1). PR is a machine-learning approach where the machine finds relevant patterns that distinguish groups of objects after being trained on examples (i.e., supervised machine learning). In contrast, the other approach to machine learning and artificial intelligence is unsupervised learning, where the machine finds new patterns without relying on prior training examples, usually by using a set of pre-defined rules. An example of unsupervised learning is clustering, where a dataset can be divided into several groups based on pre-existing definitions of what constitutes a cluster, or the number of clusters expected. A review of machine learning in the context of bioinformatics (as opposed to imaging) can be found in [3].

**Box 1. Pattern Recognition**

- PR is the task of automatically detecting patterns in datasets and using them to characterize new data. PR is a form of machine learning, which itself is a field within artificial intelligence. Machine learning can be divided into two major groups. In *supervised learning*, or PR, a computer system is trained using a set of pre-defined classes, and then used to classify unknown objects based on the patterns detected in training. In *unsupervised learning* there are no classes defined a priori, and the computer system subdivides or clusters the data, usually by using a set of general rules. An example of supervised learning is automatic detection of protein localization, in which the computer system is trained using images of probes for known sub-cellular compartments [10]. An example of unsupervised learning is clustering an expression profiling microarray experiment into groups of genes with similar expression patterns.
- Other approaches to PR include *semi-supervised learning*, which uses pre-defined classes to find new similarity relationships and define new groups, and *reinforcement learning*, in which decisions are improved iteratively based on a feedback mechanism and specified reward criteria. In this educational article we focus on the application of supervised learning to automated analysis of microscopy image datasets.

Traditionally, analysis of digital microscopy images requires identifying regions of interest (ROIs) or “objects” within the images. Once a region is isolated from the background, the resolution and dynamic range afforded by digital microscopy allows many types of measurements and statistics to be collected about the object in question, such as intensity, shape, size, and position, as well as the number of objects and their distribution [4]. This region selection can be done manually by drawing boxes or free-hand regions using an interactive tool [5], or automatically using computer algorithms known as segmentation algorithms [4], [6]. While most image analysis continues to rely on region identification, PR can also be used to process whole images or images tiled on a grid without a prior region identification step [7].

Traditional image processing is therefore predicated on the question, “can my objects of interest be identified?”. In contrast, PR is predicated on the question, “can these groups of images be distinguished?”. In this context, the input to a PR algorithm may be an entire image, a sub-image region identified with segmentation algorithms, or simply image samples in the form of rectangular tiles. Thus, in contrast to selecting and tuning a segmentation algorithm, PR requires training a computer to distinguish groups of images. These groups correspond to experimental controls, and the set of images within a group encompasses the variation within each control. Given these groups of images, the machine can learn on its own what aspects of the images represent natural experimental variation and are therefore irrelevant, and what aspects are important for distinguishing the groups of control images from each other [1], [4], [8]. This ability to sort image measurements by their relevance to a given imaging experiment allows the use of a great variety of generic image description algorithms that are not specifically related or tuned to each imaging problem, potentially making the collection of algorithms very general. The selection of algorithms and the rules for combining them are done automatically as part of the machine-learning process, eliminating the need for a microscopist to select the set of imaging algorithms to use, or adjust the parameters with which to run them.

Images taken with phase contrast, differential interference contrast (DIC), or other methods for visualizing gross morphology are notoriously difficult for computers to analyze because the perceptual model may not be visually apparent, or is challenging to encode in algorithms [9]. The types of image measurements that can be used for PR are not limited to what we can perceive, model, and encode in segmentation algorithms, making it possible to use automated methods to analyze gross morphology rather than being limited to specific probes, or a priori perceptual models.

The applicability of PR in a specific imaging experiment depends entirely (and solely) on the availability and distinguishability of control images. Thus, a PR approach to an imaging experiment is very closely tied to the biological experiment itself rather than intermediate measurements from image processing, or familiarity with the algorithms necessary to produce these measurements. PR can be used in tandem with segmentation algorithms when possible in order to exploit benefits provided by both approaches. Except in a limited sense, such as analyzing the confusion matrices of classification experiments as discussed in the *Interpreting Image Classification Output* section, most PR approaches do not yield the types of quantitative results one gets from segmentation algorithms. Instead, it can lead directly to a qualitative experimental result, such as finding the “hits” in a screen. In general, PR is useful as an exploratory imaging assay that is independent of any preconceptions of the nature or existence of morphological differences in the imaging experiment. PR requires little effort or expertise to try. It can be used to check whether morphological readouts exist and develop more specific imaging algorithms if warranted.

In this review we describe the general outline common to all PR systems used for biological microscopy, and focus on techniques and specific software packages that have been used successfully in biological image analysis. We discuss some of the requirements of the experimental setup that are necessary to take full advantage of PR and point out some of the differences between PR experiments and traditional manual evaluation of microscopy images or model-based image analysis.

## Overview of Bioimage PR Systems

**Figure 1:**
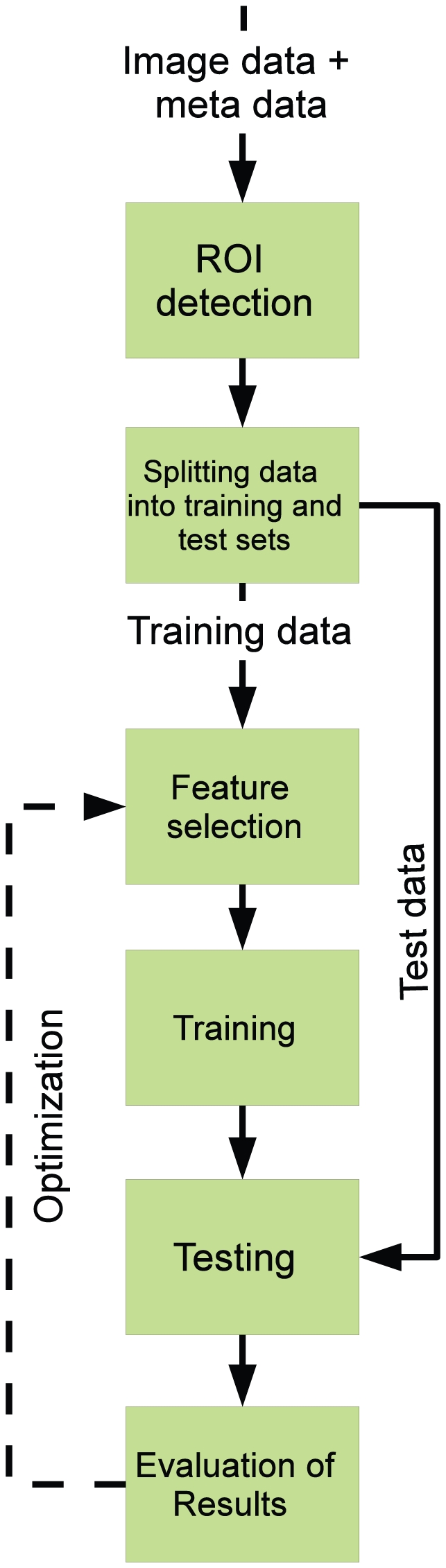
High-level architecture of bioimage analysis systems.

Although there are many examples of PR systems, the process can be summarized in several steps (Figure 1).

As with traditional image processing approaches based on object identification alone, PR can also benefit from various techniques to subdivide images into ROIs. The three principal reasons for doing so are to 1) reduce the number of pixels the PR algorithm needs to consider all at once to improve response time or increase statistical power, 2) bias the PR algorithm to process objects of interest rather than background, and 3) center or align objects that have inherent orientation. ROI detection algorithms and tools are described more thoroughly in the *Finding Regions of Interest* section.

The second step is the extraction of image content descriptors (image features), which are values that describe the image content numerically. These values can reflect various texture parameters of the image, the statistical distribution of pixel intensities, edges, colors, etc. While the dimensionality of the raw pixels can typically reach ∼1,000,000 (assuming a microscopy image of 1000
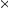
1000 pixels), the number of image features ranges between a dozen to a few hundred. While each pixel value describes the intensity at a given X,Y position, each feature value describes a specific image characteristic. A more detailed description of the types of features commonly used can be found in the *Computing Image Features* ** section.

In the next step, the image features are used to draw conclusions about the data. Generally, PR methods select features and potentially assign weights based on their ability to discriminate the classes. The refined feature set is then used to infer rules for combining them in a classifier. These two steps constitute the training stage in PR, where the goal is to correctly classify the training images. The trained classifier is then tested on control images that were excluded from the training stage. This cross-validation is important to establish the classifier's ability to identify new images, ensuring that it is not restricted to recognizing images it was trained with. More information about feature selection and classification can be found in the *Feature Selection and Classification* section.

Finally, the results of image classification need to be interpreted by the researcher in an experimental context to reach a biological conclusion. There are special considerations in this interpretation specific to PR, which is further discussed in the *Interpreting Image Classification Output* section.

## Finding Regions of Interest

As discussed in the *Overview of Bioimage PR Systems* section, the first step of computer-aided image analysis is usually to reduce the number of pixels considered by the PR algorithm. The most labor-intensive approach is to manually define regions of interest. While in some cases this can be the only option, this method introduces bias and inconsistency, and is too labor intensive to be practical for the analysis of large-scale screens. In this case, the definition of ROIs can be automated by simply dividing the image into tiles using a regular grid pattern. This simple reduction in pixel number can increase the throughput of the PR algorithms, as well as provide greater statistical power by considering a larger number of individual tiles.

In cases where objects of interest can be easily identified by segmentation algorithms (e.g., fluorescently labeled cells or structures), subsequent image analysis can be more effective if only the regions of interest are processed and analyzed, while the background areas are left out of consideration. Similarly to naive tiling, rejection of biologically irrelevant areas reduces the response time of the system. A more important role for this type of ROI detection is that it can potentially eliminate the presence of artifacts that can add noise, and degrade the efficacy of the computer analysis. In cases where the objects of interest are difficult to detect among all the objects that appear in the image, PR-based analysis can be used to “learn” which of these objects have biological meaning relevant to the experiment.

Some implementations of segmentation algorithms are designed for a specific type of object (e.g., cells), and therefore do not require intensive tuning of the system, while other tools are more general, and require adoption to the objects of interest. Widely used methods for ROI detection include global thresholding [11], watershed algorithms [12]–[14], model-based segmentation [15], and contour methods [16]. In some cases, automatic edge detection can be used to segment regions of interest [17].

A useful tool for cell segmentation is the open-source software CellTracer [18], which is written in Matlab and can be downloaded at http://www.stat.duke.edu/research/software/west/celltracer/. Another powerful tool for ROI detection is ITK (Insight Segmentation and Registration Toolkit) [19] (http://www.itk.org/), which is an open-source package designed to detect regions of interest in 2-D and 3-D microscopy images, as well as other types of biomedical imaging such as MRI and CT. VTK [20] (http://www.vtk.org/) enhances ITK with a graphical user interface. GemIdent [21] (http://www.gemident.com/) is a multi-purpose tool for detection and segmentation of objects of interest in color images, and provides an interactive graphical user interface that allows the user to tune and optimize the detection. Another tool for automatic detection of spot-like objects in microscopy images is FindSpots [22], which is based on the global thresholding method, and is capable of detecting objects in 2-D as well as 3-D images. FindSpots is available as part of the OME software package [23] (http://www.openmicroscopy.org/). sephaCe [9] (http://www.assembla.com/code/sephaCe/subversion/nodes/) is a tool that applies advanced edge and cell boundary detection to address the difficult problem of cell segmentation in brightfield images. A powerful tool for 3-D segmentation is V3D-Neuron [24], which can visualize, trace, and analyze 3-D images of neurons.

A practical approach to ROI detection is the popular ImageJ software (http://rsbweb.nih.gov/ij/). ImageJ allows the use of external plugins, which enhance it with features that are not supported by ImageJ built-in functions. Examples of ROI detection and segmentation plugins developed for the ImageJ platform include NeuronJ [25] (http://www.imagescience.org/meijering/software/neuronj/) and NeuriteTracer [26] for working with images of neurons, ITCN (http://rsbweb.nih.gov/ij/plugins/itcn.html) for finding nuclei in various cell and image types, a generic watershed segmentation algorithm [12], and many more listed at http://rsbweb.nih.gov/ij/plugins/. A project called Fiji (http://pacific.mpi-cbg.de/) repackages ImageJ along with a selection of plug-ins and other features useful for bioimage processing.

Often, objects of interest have an inherent orientation that is not preserved by the imaging system. Examples include polarized cells, as well as images of tissues and whole organisms. In these cases, the efficacy of PR can be improved if the objects of interest are registered, so that the orientation variance introduced by imaging is eliminated. In some cases, objects may differ not only in orientation, but also in scale or local geometry, requiring positional and rotational registration as well as local morphing. Combined with the required accuracy of segmentation algorithms to identify landmarks, registration can be a challenging task. Some software tools for ROI detection and segmentation, such as ITK, also offer tools for registration. An application-specific example is the registration of various images of *Caenorhabditis elegans* nematodes. Normalization of these objects for the purpose of image processing can be performed by “straightening” the worms and rotating them to a fixed orientation [27].

Sophisticated segmentation algorithms can greatly increase the signal to noise ratio, but the perceptual models they implement or the parameters used can also lead to errors. When segmentation errors are random (non-systematic), there will be a corresponding reduction in the signal to noise. Importantly though, these errors can be correlated to the experimental question, leading to systematic bias and skewing of the experimental results.

In some cases, the dataset includes a large number of ROIs, and using all of these images might severely slow down the response time of the system due to the computing resources required to compute image features for high volumes of image data, as will be explained in the *Computing Image Features* section. In these cases, to improve system response time, a subset of these ROIs can be selected randomly for classifier training. Some bioimage analysis tools also use interactive user interfaces to select the ROIs manually, and refine them based on classifier performance. This can be done iteratively, until the researcher is satisfied with the classification results. CellProfiler-Classifier provides an example of this iterative selection and classification refinement [28]. For 3-D images, objects for classification can be selected and annotated using tools such as OMERO [5], VANO [29], and V3D [24], which can allow ROI or image-based annotations of large and complex 3-D microscopy images, including combinations of 3-D, multi-channel, and time-lapse.

It is important to note that when using segmentation software developed independently of PR, a degree of integration with PR-based software tools is required. Often, the segmentation results can be exported as separate images that can be straightforward to transfer to PR software for subsequent analysis. In other cases, this integration requires scripting or substantial programming. Therefore, experimentalists are encouraged to first consider software packages developed specifically for PR in microscopy images, providing a full start-to-finish solution. These comprehensive software tools are described in the *Software Tools* section.

## Computing Image Features

After reduction of the image size by ROI selection, raw pixel data is usually still not appropriate for direct processing by PR algorithms. Instead, the pixel data is further summarized for its “image content” using a set of feature extraction algorithms. Each algorithm reads image pixels and outputs one or more numerical values that describe various aspects of the image. These algorithms are usually very general in that they can operate on any set of pixels without specifying any parameters. The types of image features vary depending on the PR software package, but generally consist of texture descriptors, statistical distribution of pixel values, shape and edge features, coefficients of polynomial expansions representing the image, and others.

Since there is no “typical” microscopy image or experiment, and the types of image content descriptors are virtually unlimited, there is no standard set of feature extraction algorithms. Additionally, different image features can be relevant to different experiments—even when they are based on the same types of images. Finally, image features calculated by computers can be almost arbitrarily detailed, and can describe patterns that people cannot readily perceive. The ability to detect differences between images automatically without the requirement for a pre-conceived perceptual model is an important advantage of PR for bioimage informatics. Therefore, in most bioimage PR systems, a larger set of image content descriptors is computed than is ultimately used after feature selection and training. This is done to cover a variety of possible morphological aspects of the images and preserve the generality of the approach [10], [28], [30].

As low-level image features are not related to any specific imaging problem, they have little utility outside of PR. Thus, modules for computing image features are normally a part of broader applications, and are not distributed as independent software products. Higher-level tools such as CellProfiler [31], wndchrm [32], and Protein Subcellular Location Image Database (PSLID) [33] apply image feature extraction as an intermediate step, but the values can be exported to third party tools.

## Feature Selection and Classification

After the image features are computed for all images in the dataset, the samples can be classified or assessed for similarity by using PR tools. Image classification is a task in which the computer system automatically assigns images to one of several user-defined image classes. An image class is simply a collection of images from an experimental control. Classification is normally done by first splitting the dataset into training and test image pools. The training data are used to automatically define the classification rules, and the test data are used to assess the effectiveness of these rules, and their ability to consistently reflect the data. Typically, several training/testing experiments are done automatically by randomly splitting the dataset and running multiple trials, as described in the *Experimental Considerations for Effective PR* section.

Many of these image features are expected to be irrelevant to the specific imaging problem being considered and contribute only to noise, while others contribute varying degrees of discriminative power to the classifier. Selection of relevant features is generally performed automatically using one of the methods described below. The automated computation and selection of image features without user intervention or parameter tuning is a key factor allowing robust automation of PR and its adaptability to virtually any image type.

There are two major approaches to feature selection: *filters* and *wrappers*. The filtering approach typically uses statistical methods to process the entire set of features to select those most informative. The selection is independent of the classifier ultimately used in the imaging experiment, and thus the features selected are not specific to the downstream classifier. Wrapping, in contrast, is based on selecting subsets of features by testing them in a classifier. Thus, wrapping can select features specific to the downstream classifier being employed.

A simple example of the filtering approach is computing the Fisher score for each feature, and rejecting a certain percentage of the features with the lowest scores. The Fisher score is a ratio of the variance in the feature value between classes to its variance within classes, giving features with high discriminative power higher scores. This approach is implemented in the wndchrm image analysis tool (available at http://ome.grc.nia.nih.gov/wnd-charm/), where the Fisher scores are also used as feature weights. While providing accurate results for a variety of image types [34], a potential downside of this method is feature redundancy due to correlations in feature values between seemingly unrelated feature extraction algorithms. Selecting more than one image feature from a group of inter-correlated features will not add to the overall effectiveness of the feature set, and may contribute to noise by over-representing a certain type of image content. An effort to address this issue directly is the minimum redundancy maximum relevance (mRMR) algorithm [35], available at http://penglab.janelia.org/proj/mRMR/.

Another approach is *remapping*, where the original feature space is substituted with another of lower dimensionality and possibly improved separability. A common transformation used in several areas of bioinformatics is principal component analysis (PCA) that has been successfully used for the analysis of DNA microarray data [36], and has also been applied to image feature reduction [37]. PCA maps the feature space into a smaller number of mutually orthogonal principal components. While the primary criterion of PCA is preservation of data variance, other techniques have been proposed that use different principles for the purpose of dimensionality reduction. One such family of methods is *manifold learning*, where a manifold [38] is assumed to be embedded in the higher dimensional feature space. Determining this manifold effectively implements a non-linear transformation into a smaller sub-space. There are several additional algorithms and implementations for these transforms, including isomap [39], local linear embedding [40], graph embedding [38], and others. Unfortunately, the public availability of software that use these techniques is currently lagging, and where available, the software requires additional programming to be practically useful. Examples of implementations available include Matlab libraries for ISOMAP (http://isomap.stanford.edu/), a demonstration example of several manifold algorithms with a graphical user interface (http://www.math.ucla.edu/~wittman/mani/), as well as a Matlab toolbox for dimensionality reduction containing a mixture of linear and non-linear algorithms (http://ict.ewi.tudelft.nl/~lvandermaaten/Matlab_Toolbox_for_Dimensionality_Reduction.html). Several feature selection and transformation techniques for identifying sub-cellular organelles are compared in [37], but to date, a systematic analysis of manifold learning approaches applied to biological imaging problems has not been attempted.

*Wrapping* selects features based on their actual performance in the classifier, in many cases providing better feature selection and greater classification accuracy than filtering. It should be noted that in most cases filtering is significantly faster than wrapping, which relies on running many iterations with different subsets of the feature bank. A collection of several wrapping methods is available through the ToolDiag software suite, that can be downloaded at http://sites.google.com/site/tooldiag/. Another useful tool for feature selection and classification is RapidMiner [41] (http://www.rapidminer.com/), which provides various feature selection and classification algorithms with a graphical user interface environment. WEKA [42] is another open-source utility that provides a rich set of classification and feature selection tools, and can be downloaded at http://www.cs.waikato.ac.nz/ml/weka/.

Classifier training automatically deduces rules for combining the most informative features into a trained classifier that can be used to associate unknown images with the user-defined classes. One of the simplest types of classifiers is nearest-neighbor, where the class of the unknown image is determined from the training image with the most similar feature values. WND (a part of WND-CHARM [30]), is a variation of this approach where the training images are used to model a probability distribution for each class. One of the first applications of PR classifiers to biological imaging [10] used neural networks for identifying sub-cellular organelles. The current implementation of this approach is in PSLID [33]. Another classification approach is GentleBoosting [43], which is used by CellProfiler [28].

More recently, support vector machines (SVMs) [44] have become popular in biological image processing as well as PR in general [45]. This type of classifier is used in Enhanced CellClassifier [46], as well as in an analysis of drug response in single cells [47]. An implementation of SVM is SVM*^light^* [48] (http://svmlight.joachims.org/), and SVM*^perf^* [49], which can be downloaded at http://svmlight.joachims.org/svm_perf.html. These tools also provide a user interface and can be used as independent tools, as opposed to some other available SVM libraries such as LIBSVM [50], which are meant to be integrated into other programs and thus require programming skills. Another useful software package that offers a wide selection of classification methods is the ToolDiag PR toolbox.

Most classifiers reported in the literature are tested using a relatively low number of classes, typically not more than a few dozen. Biological ontologies, however, can extend to thousands of terms, and if they are used as the basis for classification, the number of classes can increase dramatically [51]–[53]. One approach to working with large numbers of classes is to reformulate the classification problem as a system of classifiers, each operating on a small set of classes [54]. Some types of classifiers [30] do not appear to be negatively affected by even a thousand classes (L. Shamir, unpublished data using the FERET dataset from NIST, consisting of 994 classes; [55]). In some cases, it is also possible to exploit estimates of class similarity (see the *Interpreting Image Classification Output* section below) to cluster a large set of classes into a smaller subset [7], [32].

## Interpreting Image Classification Output

The most basic piece of information obtained when validating a classifier is the classification accuracy. Determining this requires reserving a pool of test images that were not used for training, but whose class is known. Classification accuracy is measured by the number of test images that were classified correctly, divided by the total number of images that the classifier attempted to classify. This number reflects the ability of the classifier to accurately associate a test image with its correct class. Clearly, a higher classification accuracy indicates that the image classifier is more informative, and can discriminate between images that belong in different classes. However, as explained in the *Experimental Considerations for Effective PR* section, the classification accuracy itself sometimes does not have any biological meaning, and can lead to false conclusions unless analyzed carefully and tested against the appropriate controls.

In binary classification, the accuracy is often reported in terms of *sensitivity* and *specificity*, which are commonly used in disease diagnosis. The sensitivity of a classifier is defined as the proportion of true positives that were correctly detected by the classifier as positives, and the specificity is defined as the proportion of the negatives that were correctly classified as negatives. Other performance metrics for binary classifiers include the false positive rate (FPR) and the false negative rate (FNR). A thorough discussion about performance metrics for binary classifiers can be found in [56].

A more informative output of a classifier validation experiment is the confusion matrix (see Table 1). Each cell contains the number of test images known to be members of the class specified by the row label that were classified as the class specified by the column label. The number of correctly classified images for each class is found on the diagonal, and the cells off of the diagonal report mis-classifications. Thus, the overall classification accuracy is the total in the diagonal cells divided by the total in all of the cells.

**Table 1:** Confusion Matrix for Classifying H&E-Stained Mouse Liver Sections by Age.

	1 Month	6 Months	16 Months	24 Months
1 month	**245**	22	43	10
6 months	218	**719**	117	66
16 months	47	18	**225**	30
24 months	73	99	227	**705**

The confusion matrix also allows estimating the degree of similarity between classes. For instance, if the confusion matrix shows that the image classifier has a high degree of confusion between a pair of classes in a given row, it is an indication that these two classes are more similar to each other than the other classes in the row. These similarities can have interesting biological implications. Depending on the classifier and the imaging problem, however, these similarities can also be a property or limitation of the classifier itself. Therefore these types of similarities need to have independent confirmation—either biological, or using a different approach to PR, and ideally both.

In some cases, the differences between the classes reflect an inherent order. For instance, if each successive class is a treatment with an increasing dose, or a time course as in Table 1, the confusion between neighboring pairs of cells in a row is expected to be higher than cells farther apart. In this case, the highest value of each row in the confusion matrix is expected to be on the diagonal, and the other values in each row should decrease for cells further away from the diagonal.

## Experimental Considerations for Effective PR

Experiments that utilize PR for microscopy image analysis introduce several important considerations. As described in the *Computing Image Features* and **Feature Selection amd Classification** sections, the image features are selected automatically by their discriminative power, and the classification rules are determined by the system. Therefore, if two sets of images have biologically irrelevant differences between them (i.e., due to systematic errors), the PR analysis could classify the two sets accurately, but the classification would be based on artifacts. Image analysis using PR can discriminate between images taken using different microscopes, objectives, cameras, etc., and potentially lead to false conclusions. In addition, it can also discriminate images taken by different experimentalists. For example, if two different treatments are studied, and images for each treatment are collected by a different person, the experimenter's acquisition parameters (which could be subjective) can lead to a detectable difference between the sets of images where no biological difference exists.

The potential for observer bias skewing PR results means that little or no quality control should be done during manual acquisition or subsequent to automated acquisition. Traditionally, images are selected manually for being representative of the biological treatment. In contrast, when applying PR, it is the entire set of images for a particular treatment that represent the class, rather than individual images. Manual selection of images introduces considerable observer bias, which may skew the PR results.

Since image analysis using PR is sensitive to artifacts, image collection should be as consistent as possible to reduce the number of non-biological differences. For this reason, it is important to collect control images in every session of image collection or for each experimental batch. Control images can be images of subjects that do not reflect any biological differences (e.g., untreated cells). If the classifier is able to differentiate between the sets of control images from the different sessions or experimental batches, the analysis may be affected by artifacts. If the classifier is not able to differentiate between the sets of control images, but can classify between the different treatments, then it can be deduced that the different treatments are reflected in the image content.

Consider a classifier that can differentiate between biologically equivalent controls as well as between treatments. For example, an accuracy of 55% between controls compared to 85% between treatments indicates that though systematic errors are present, the biological signal predominates. Here, the relative classification accuracy between the two can be compared and the classification result can be accepted because the difference in accuracy is sufficiently great. A better approach is to make unavoidable systematic bias non-systematic. For example, if two researchers must collect data, it is better for each researcher to collect the entire set of treatments so that their data can be equally pooled into classes for classifier training. The effectiveness of this approach can be confirmed experimentally by testing the classification accuracy of acquisition controls from the different experimenters pooled together.

Image classifiers differentiate image classes based on the strongest morphological signal, which for various reasons may not be of interest to the experimenter. An example of this is a cell growth effect that is not of interest combined with a morphological effect that may be of greater interest. One option for eliminating the growth effect is to use segmentation to identify individual cells followed by PR on classes composed of balanced cell numbers. When segmentation is not possible or undesirable, an alternative is to force the classifier to disregard effects that are considered unimportant. One example of this was discussed above, where data collected by different researchers is mixed together in each of the defined classes. An undesired growth effect can similarly be eliminated from consideration by defining each experimental class using several different cell densities. A third option was used by our group to reduce variation between experimenters [57], as well as eliminating recognition of individual mice when analyzing the gender or age of liver sections [58]. Here, we trained a classifier to discriminate classes composed of the artifact we wanted to eliminate (i.e., images collected by one experimenter versus images collected by someone else; liver sections from individual mice to train a one mouse per class classifier). We eliminated the undesired classification signal from the experimental classifier by subtracting the feature weights of the artifact classifier from the experimental one. For mouse livers, we were able to show that this corrected classifier could resolve gender equally well, but could no longer identify individual mice [58]. Similarly, using this approach to eliminate a growth effect would involve training an artifact classifier composed of classes with different cell densities, where each class contained the full range of experimental effects. This type of correction is highly dependent on the type of classifier being used, and is not feasible in most types of classifiers.

When testing a classifier for its ability to differentiate between sets of images, the classification accuracy should be measured in several runs, where different images are used for training and testing in each run. These multiple trials test whether the classifier's performance is overly dependent on the specific images used in training. When the number of control images is extremely limited, validation can also be performed in a “leave one out” (or round-robin) manner, where training is performed using all but one of the images, and the left-out image is used to validate the classifier. This is normally systematically repeated, such that each image in the dataset is tested in turn.

It should also be noted that it is important to have the same number of training images in each class to avoid potential bias caused by an unbalanced image distribution. If the classifier was capable only of random guessing, then it should assign test images to the defined classes with equal probability. If one of the training classes was much larger than the others, a classifier may assign test images to the larger class at a rate higher than expected for random guessing, while the smaller classes would be assigned with a less-than-random probability. There are several mechanisms that could lead to this result, and some classifiers are more prone to this bias than others. The safest approach is to use the same number of images in each class for training. If this is not practical, a negative control experiment can reveal if the classifier suffers from this bias. In this case, the class assignments of the training images should be randomly scrambled, and the resultant classifier should be checked to report the expected random distribution of class assignments.

While it is important to train a classifier using an equal number of images per class, using the same number of test images can also be important to obtain an unbiased assessment of classifier performance. For instance, if a classifier of two classes has 40 test images of class *A* and 10 test images of class *B*, correct classification of all class *A* images will lead to an accuracy of 80%, even if the classifier misclassified all test images of class *B*. These results might mislead the experimentalist to believe that the classifier is performing adequately, even though it classifies all images as class *A*. Another approach to address this problem is to measure the mean classification accuracy for each class separately [32] rather than relying solely on the overall percentage of images that were classified correctly. For instance, in the case above, the 100% accuracy of the test images of class *A* will be balanced by the 0% accuracy of class *B*, providing a per-class average classification of 50%, clearly indicating that the classifier does not work.

The image classifier must be trained with a sufficient number of sample images for each of the predefined classes. Therefore, an experiment that is based on PR requires a significantly larger number of images than an experiment in which the conclusions are made by manual inspection or by the use of segmentation tools alone. Normally, accuracy increases as the training set gets larger, eventually reaching a plateau where the classifier is said to be “saturated”. This number can be determined empirically by running the classifier repeatedly with different numbers of training images, plotting the classification accuracy against the number of training images. This classifier analysis can also be used to determine whether poor classification performance is due to insufficient training images, or due to the classes being indiscernible by the chosen classifier.

The number of training images required for accurate classification can vary depending on the difficulty of distinguishing the classes, and the variability within each class. In our experience with wndchrm, if the classes are easily distinguishable by eye and the images within classes are visually consistent, generally no more than a dozen images are required for training. An extreme example is identifying binucleate phenotypes. Here a classification accuracy of 98% can be achieved using a single training image. In contrast, our study of *C. elegans* muscle degeneration throughout lifespan [57] used 85 training images for each of seven classes, and could have used more. In this case, human observers could reliably distinguish only very young worms from very old ones. In cases where smaller training sets can provide reasonable performance, using larger training sets was not found to be deleterious.

## Software Tools

While there are numerous publicly available stand-alone software tools that can perform specific tasks in the process of PR-based image analysis such as segmentation, feature selection, classification, etc., using these together may require programming skills for their integration. Fortunately, some software packages have been developed to provide a start-to-finish solution for bioimage analysis and HCS, and are often equipped with user-friendly graphical user interfaces targeted at bench biologists. Unfortunately, not all PR software is well integrated and user friendly, and in these cases some additional help should be sought from bioinformaticians, or the growing number of biologists with significant expertise in computing and information technology.

The software discussed in this section was selected based on four parameters: usability without further software development, integration of PR techniques discussed above, an established user community, and open-source code. Although availability of source code would seem to be of little consequence to non-programmers, it is an important consideration. The foremost reason scientifically is that at least in principle, the implementation of the algorithms by the software is independently verifiable. There are also practical considerations. If the original authors abandon the software project without providing the source code, then the software may soon stop running on new versions of operating systems and hardware. If the software was an integral part of the processing pipeline, then previous experiments may need to be repeated with a new software package in order to compare them to new results. The availability of source code usually also means that there is a widely distributed pool of experts that can modify the software or just keep it updated. Even when this pool doesn't exist, a professional programmer can be hired to fix, modify, or update the software if this becomes necessary.

In this section we mention one of the most popular image processing programs, ImageJ, and discuss four complete systems for biological imaging that rely on PR techniques: CellProfiler-Classifier, PSLID, wndchrm, and CellExplorer, listed in Table 2. Although ImageJ is not specifically designed for PR, there are many plug-ins available for segmentation, which can be valuable for data reduction prior to classification as discussed above. Manipulation of image groups is an integral part of analysis by PR, and ImageJ does not provide a mechanism for associating images with each other. The grouping of images would allow consequent grouping of the ROIs produced by various segmentation plug-ins. A PR plug-in could then use these multi-image ROIs to define classes for training. There are several scripts available for batch processing images together in ImageJ, so the notion of image groups may be implemented in the future.

**Table 2:** Publicly Available Image Analysis Software Tools Employing or Useful for PR in Biological Microscopy.

Tool	ROI Detection	Classification	Graphical User Interface	Open Source	Language	Platforms	Required Software	Microscopy	Web Site
ImageJ	Yes(plugin)	n.a.	Yes	Yes	Java	Linux, MacOSWindows	None	All	http://rsbweb.nih.gov/ij/
PSLID/SLIC	Yes	ANN, SVM	No	Yes	Matlab, C, Python	Linux	Postgres, tomcatMatlab	Fluorescence	http://pslid.cbi.cmu.edu/release/
CellProfiler	Yes	GentleBoosting	Yes	Yes	Python, Matlab	Linux, MacOSWindows	None	Fluorescence	http://www.cellprofiler.org/
wndchrm	No	WND	No	Yes	C	Linux, MacOSWindows	None	All	http://ome.grc.nia.nih.gov/wnd-charm/
CellExplorer	Yes	SVM	Yes	Yes	Matlab	Linux, MacOSWindows	Matlab	Confocal/3-D	http://penglab.janelia.org/proj/cellexplorer/

The PSLID [33] was the first application of PR for microscopy images [59]. This project aims to eventually enumerate and discern all subcellular localization patterns. Even though this may seem quite specialized, localization is not limited to identifying organelles, but can be used to describe any type of subcellular distribution for a protein, stain or other biomarker. PSLID has evolved over the years to analyze patterns in multiple fluorescence channels as well as in 3-D and over time. PSLID can be used with a database to manage large image collections. A full installation can also include a Web service to perform image-based or localization-based searches.

The goal of the CellProfiler project is to provide a user-friendly image processing environment for HCS [60]. In HCS, high-resolution imaging of cells is used as an assay in a screen of chemical compounds or RNAi libraries. These experiments easily involve tens or hundreds of thousands of images, where manual scoring is impractical. Similarly to ImageJ, CellProfiler includes tools to identify (segment) cells and nuclei, and report various statistics on the objects found in an image. In contrast to ImageJ, CellProfiler is designed around image processing pipelines, where many thousands of images can be analyzed in batch. The recent addition of CellProfiler-Classifier [28] introduces PR techniques to classify cells into user-defined phenotypes. Interestingly, CellProfiler-Classifier can also find cells that do not fit into any of the predefined phenotypes, making it useful for identifying rare or low-penetrance phenotypes. Similarly to PSLID, CellProfiler places constraints on how the imaging experiment is conducted—mainly that the cells must be easily identifiable by the segmentation algorithms used. Generally this requires staining with a fluorescent cytoplasmic marker as well as a fluorescent nuclear marker in addition to any markers being used in the actual experiment. In light of these considerations, the work done with CellProfiler and PSLID thus far is limited to fluorescence microscopy. In contrast to PSLID, CellProfiler is meant to be used on a user's desktop rather than provide a centralized Web-based service, so it is somewhat easier to install and use.

The WND-CHARM [30] project was initiated to provide PR tools for the analysis of a broad variety of image types, and provide a means to explore different classifiers trained by grouping the same images in various ways. This software is a command-line tool [32] that processes images arranged into folders representing the image classes, and produces reports in HTML format that are viewable in any Web browser. Unlike PSLID and CellProfiler, WND-CHARM has been extensively tested on a variety of image types, including phase-contrast, differential-interference contrast, and histological stains, as well as fluorescence microscopy [34]. Other than dividing the images into tiles, WND-CHARM does not supply any segmentation tools of its own, although any software that can produce cropped images of segmented cells can be used to provide images to WND-CHARM. Despite the lack of segmentation tools, WND-CHARM has been shown to accurately score imaging assays that have been traditionally analyzed by segmentation algorithms, such as scoring a screen for binucleate phenotypes with 100% accuracy [34]. WND-CHARM is self-contained and does not rely on extensive external math software such as Matlab, or database infrastructure such as Oracle and MySQL. This portability allows it to be easily integrated into other software that provides segmentation and database functionalities.

Another useful tool for automatic analysis of biological images is CellExplorer [61]. CellExplorer was designed for the analysis of *C. elegans* images, but was also found effective for other model organisms such as drosophila. The package includes advanced segmentation, annotation and straightening algorithms [62] of 3-D microscopy images. The segmented nuclei can also be classified automatically using an SVM classifier. CellExplorer is freely available for download; however, it requires the installation of Matlab, which is commercial software.

The software tools described above and the segmentation tools described in the *Finding Regions of Interest* section can be tested using publicly available biological image datasets, which include images of different organisms acquired using different types of microscopy, magnifications, etc. Some useful publicly available image datasets include the PSLID datasets (http://murphylab.web.cmu.edu/data/), the IICBU benchmark suite [34] (http://ome.grc.nia.nih.gov/iicbu2008/), and the Broad Bioimage Benchmark Collection http://www.broadinstitute.org/bbbc/.

## Discussion

In this review we describe the basic concepts, terminology and software tools for PR-based imaging assays for biology. The information provided is directed towards the bench scientist looking for an alternative to traditional image processing approaches. Although most of the current applications of PR are used for analyzing very large image datasets (e.g. PSLID, CellProfiler-Classifier [28], [33]), these techniques can be applied just as easily to more conventional imaging assays performed in non-specialist laboratories. The principal advantage of the approach is its potential for processing a broad variety of image types without requiring customized software or parameter tuning for each imaging experiment.

The ability to compare images to each other regardless of image type can lead to the discovery of new knowledge from existing data. General sequence comparison algorithms such as BLAST have transformed the archiving and retrieval of sequence data in public repositories such as GenBank into a field (genomics) where new knowledge is routinely synthesized from existing sequence collections. By analogy, the integration of generalized image comparison algorithms with large, diverse, and well-annotated public image repositories is an essential step toward more complete data extraction from biological images. For example, metadata fields used to annotate images either manually or by using specialized algorithms can serve as the basis for defining training classes for PR algorithms. The resulting classifiers can then annotate images where these fields haven't been defined. While this process can be fully automated, the tools developed for this approach can also be used interactively to pose questions about potential new relationships within these image collections.

Although the techniques outlined in this review can lead to general image comparison algorithms, image data poses several challenges that are only now beginning to be addressed: quantitative image comparisons within multiple contexts, relevant ranking algorithms, and integrated image repositories. Context can be understood in text searching by considering the query “Orange”. The results of this query will depend on whether the context is colors, fruit, computer companies, or cellular service providers. This level of ambiguity is typical for image-based queries where every image can be viewed in several distinct contexts. The practical implication for PR is that a given image in a repository may be used for training several different classifiers, or alternatively a group of unrelated classifiers may analyze an image along several distinct contexts. Search interfaces typically allow only a limited specification of context if they allow one at all (e.g., Google Images, Videos, Maps, News, etc.). This is not adequate for image-based search due to the high degree of ambiguity in the search context, the difficulty of defining an implied context, and its complete dependence on the experimental question being asked.

A relevant ranking algorithm is a key characteristic of a useful search interface because the results most relevant to the user are presented first. In scientific image-based searching, the ranking of search results should be based on image similarity measured along one or more biologically relevant contexts, specified by the user. Bisque and PSLID provide image-based search algorithms that return sets of images from the database that are most similar to the query image. However, it is not possible to refine the search context (i.e., similar in what way?), and the biological relevance of the result ranking cannot be easily evaluated. Quantitative image similarity is an important tool for imaging assays such as dose-response, time courses, and comparisons of phenotypes. In addition, measures of similarity can form the basis for clustering algorithms, where new groupings of images can be discovered based on existing or related contexts using objective statistical criteria. PR techniques have been previously used to quantify image similarity within an experimental context. For example, a mis-classification rate was used to measure cellular response to varying drug doses [47], and a direct measurement of similarity using a linear classifier was used to measure sarcopenia in the *C. elegans* pharynx [57]. Currently, direct measurements of contextualized image similarity using general PR techniques is an area of active and ongoing research.

Ultimately, for image informatics to mature, it requires both contextual image comparison algorithms and fully integrated image repositories. The last decade has seen the creation of several specialized image repositories, some examples of which include the Visible Human Project (http://www.nlm.nih.gov/research/visible/visible_human.html), the Biomedical Informatics Research Network (BIRN, http://www.birncommunity.org/), the cancer Biomedical Informatics Grid (caBIG, https://cabig.nci.nih.gov/), the JCB Data Viewer (http://jcb-dataviewer.rupress.org/), and a new initiative called The Cell: An Image Library (http://cellimagelibrary.org/). Currently, these are collections of images that can be searched solely by their annotations and used to exemplify various biological processes and features. The software infrastructure for these image repositories has matured in parallel over the past decade from early projects such as OME [63],[64] and PSLID [33] to projects like Bisque [65] and OMERO [5] that are maintained by full-time developers and used by an increasing number of imaging labs. These types of image data management systems are primarily concerned with the definition and structure of imaging metadata, and provide interfaces for annotation, search, and browsing. Furthermore, these systems allow queries based either on comparisons between entered text and textual annotations in the database, or image features extracted from a query image compared to those of the archived images. The integration of image repositories like these with universal, contextualized image comparison algorithms will substantiate the premise of image informatics: that pre-existing image datasets can be analyzed in-silico to find new relationships between images, leading to new knowledge and discovery.

## References

[1] Peng H (2008). Bioimage informatics: a new area of engineering biology.. Bioinformatics.

[2] Jones TR, Carpenter AE (2007). Open-source software automates high-throughput imaging.. BioPhotonics.

[3] Tarca AL, Carey VJ, Chen X-w, Romero R, Draghici S (2007). Machine learning and its applications to biology.. PLoS Comput Biol.

[4] Ljosa V, Carpenter AE (2010). Introduction to the quantitative analysis of two-dimensional fluorescence microscopy images for cell-based screening.. PLoS Comput Biol.

[5] Swedlow JR, Goldberg IG, Eliceiri KW (2009). Bioimage informatics for experimental biology.. Annu Rev Biophys.

[6] Fuller CJ, Straight AF (2009). Image analysis benchmarking methods for high content screen design.. J Microsc.

[7] Shamir L, Eckley DM, Delaney J, Orlov N, Goldberg IG (2009). An image informatics method for automated quantitative analysis of phenotype visual similarities.. IEEE NIH Life Sci Syst Appl Workshop.

[8] Coelho LP, Glory-Afshar E, Kangas J, Quinn S, Shariff A (2010). Principles of bioimage informatics: focus on machine learning of cell patterns.. Lect Notes Comput Sci.

[9] Gooding AR, Christlieb M, Brady M (2008). Advanced phase-based segmentation of multiple cells from brightfield microscopy images.. Proc IEEE 5th Int Symp Biomed Imaging.

[10] Boland MV, Murphy RF (1999). Automated analysis of patterns in uorescence-microscope images.. Trends Cell Biol.

[11] Sahoo PK, Soltani S, Wong AKC (2008). Survey: a survey of thresholding techniques.. Comput Vis Graph Image Process.

[12] Vincent L, Soille P (1991). Image-proc skeleton segmentation.. IEEE Trans Pattern Anal Mach Intell.

[13] Lindeberg T (1993). Detecting salient blob-like image structures and their scales with a scale-space primal sketch: A method for focus-of-attention.. Int J Comput Vis.

[14] Roerdink BTM, Meijster A (2001). The watershed transform: Definitions, algorithms and parallelization strategies.. Fundam Inform.

[15] Cong G, Parvin B (2000). Model-based segmentation of nuclei.. Pattern Recognit.

[16] Vromen J, McCane B (2006). Red blood cell segmentation using guided contour tracing.. 18th Proc Annu Colloq Spatial Inform Res Centre.

[17] Li G, Liu T, Nie J, Guo L, Chen J (2008). Segmentation of touching cell nuclei using gradient flow tracking.. J Microsc.

[18] Wang Q, Niemi J, Tan CM, You L, West M (2009). Image segmentation and dynamic lineage analysis in single-cell uorescent microscopy.. Cytometry A.

[19] Yoo T, Metaxas D (2005). Open science - combining open data and open source software: Medical image analysis with the Insight Toolkit.. Med Image Anal.

[20] Schroeder W, Martin K, Lorensen B (2006). The visualization toolkit: an object-oriented approach to 3D graphics. 4th edition.

[21] Kapelner A, Lee P, Holmes S (2007). An interactive statistical image segmentation and visualization system.. MEDIVIS.

[22] Platani M, Goldberg I, Swedlow JR, Lamond AI (2000). In vivo analysis of Cajalbody movement, separation, and joining inlive human cells.. J Cell Biol.

[23] Schiffmann DA, Dikovskaya D, Appleton PL, Newton IP, Creager DA (2006). Open Microscopy Environment and FindSpots: integrating image informatics with quantitative multidimensional image analysis.. BioTechniques.

[24] Peng H, Ruan Z, Long F, Simpson JH, Myers EW (2010). V3D enables real-time 3D visualization and quantitative analysis of large-scale biological image data sets.. Nat Biotechnol.

[25] Meijering E, Jacob M, Darria P, Hirling H, Unser M (2004). Design and validation of a tool for neurite tracing and analysis in uorescence microscopy images.. Cytometry.

[26] Pool M, Thiemann J, Bar-Or A, Fournier AE (2008). NeuriteTracer: A novel ImageJ plugin for automated quantification of neurite outgrowth.. J Neurosci Methods.

[27] Peng H, Long F, Liu X, Kim S, Myers E (2008). Straightening C. elegans images.. Bioinformatics.

[28] Jones TR, Carpenter AE, Lamprecht MR, Moffat J, Silver SJ (2009). Scoring diverse cellular morphologies in image-based screens with iterative feedback and machine learning.. Proc Natl Acad Sci U S A.

[29] Peng H, Long F, Myers EW (2009). VANO: a volume-object image annotation system.. Bioinformatics.

[30] Orlov N, Shamir L, Macura T, Johnston J, Eckley DM (2008). WND-CHARM: Multi-purpose image classification using compound image transforms.. Pattern Recognit Lett.

[31] Lamprecht MR, Sabatini DM, Carpenter AE (2007). CellProfiler: free, versatile software for automated biological image analysis.. BioTechniques.

[32] Shamir L, Orlov N, Eckley DM, Macura T, Johnston J (2008). Wndchrm an open source utility for biological image analysis.. Source Code Biol Med.

[33] Boland MV, Murphy RF (2001). A neural network classifier capable of recognizing the patterns of all major subcellular structures in uorescence microscope images of HeLa cells.. Bioinformatics.

[34] Shamir L, Orlov N, Eckley DM, Macura T, Goldberg IG (2008b). IICBU 2008: a proposed benchmark suite for biological image analysis.. Med Biol Eng Comput.

[35] Ding C, Peng H (2005). Minimum redundancy feature selection from microarray gene expression data.. J Bioinform Comput Biol.

[36] Knudsen S (2002). A Biologist's Guide to Analysis of DNA Microarray Data.

[37] Huang K, Velliste M, Murphy RF (2003). Feature reduction for improved recognition of subcellular location patterns in uorescence microscope images.. Proc SPIE.

[38] Basavanhally AN, Ganesan S, Agner S, Monaco JP, Feldman MD (2010). Computerized image-based detection and grading of lymphocytic infiltration in HER2+ breast cancer histopathology.. IEEE Trans Biomed Eng.

[39] Tenenbaum JB, de Silva V, Langford JC (2000). A global geometric framework for nonlinear dimensionality reduction.. Science.

[40] Roweis ST, Lawrence KS (2000). Nonlinear dimensionality reduction by locally linear embedding.. Science.

[41] Mierswa I, Wurst M, Klinkenberg R, Scholz M, Euler T (2006). YALE: rapid prototyping for complex data mining tasks.. KDD.

[42] Holmes G, Donkin A, Witten IH (1994). Weka: A machine learning workbench.. ANZIIS.

[43] Friedman JH, Hastie T, Tibshirani R (2000). Additive logistic regression: a statistical view of boosting.. Ann Stat.

[44] Vapnik VN (1995). The nature of statistical learning theory.

[45] Ben-Hur A, Ong CS, Sonnenburg S, Scholkopf B, Ratsch G (2008). Support vector machines and kernels for computational biology.. PLoS Comput Biol.

[46] Misselwitz B, Strittmatter G, Periaswamy B, Schlumberger MC, Rout S (2010). Enhanced CellClassifier: a multi-class classification tool for microscopy images.. BMC Bioinformatics.

[47] Loo LH, Wu LF, Altschuler SJ (2007). Image-based multivariate profiling of drug responses from single cells.. Nat Methods.

[48] Joachims T (2000). Estimating the generalization performance of a SVM efficiently.. Proc Int Conf Mach Learn.

[49] Joachims T (2006). Training linear SVMs in linear time.. KDD.

[50] Fan RE, Chen PH, Lin CJ (2005). Working set selection using the second order information for training SVM.. J Mach Learn Res.

[51] Carpenter AE, Sabatini DM (2004). Systematic genome-wide screens of gene function.. Nat Rev Genet.

[52] Carson JP, Ju T, Lu HC, Thaller C, Xu M (2005). A digital atlas to characterize the mouse brain transcriptome.. PLoS Comput Biol.

[53] Peng H, Long F, Eisen M, Myers E (2006). Clustering gene expression patterns of y embryos.. Proc IEEE Int Symp Biomed Imaging.

[54] Zhou J, Peng H (2007). Automatic recognition and annotation of gene expression patterns of fly embryos.. Bioinformatics.

[55] Phillips PJ, Moon H, Rauss PJ, Rizvi S (2000). The FERET evaluation methodology for face recognition algorithms.. IEEE Trans Pattern Anal Mach Intell.

[56] Gardner MJ, Altman DG, Gardner MJ, Altman DG (1989). Calculating confidence intervals for proportions and their differences.. Statistics with confidence.

[57] Johnston J, Iser WB, Chow DK, Goldberg IG, Wolkow CA (2008). Quantitative image analysis reveals distinct structural transitions during aging in Caenorhabditis elegans tissues.. PLoS ONE.

[58] Macura TJ (2009). Automating the quantitative analysis of microscopy images [PhD thesis].

[59] Boland MV, Markey MK, Murphy RF (1998). Automated recognition of patterns characteristic of subcellular structures in uorescence microscopy images.. Cytometry.

[60] Jones TR, Kang IH, Wheeler DB, Lindquist RA, Papallo A (2008). CellProfiler Analyst: data exploration and analysis software for complex image-based screens.. BMC Bioinformatics.

[61] Long F, Peng H, Liu X, Kim SK, Myers EW (2009). A 3D digital atlas of C. elegans and its application to single-cell analyses.. Nat Methods.

[62] Peng H, Long F, Liu X, Kim SK, Myers EW (2008). Straightening Caenorhabditis elegans images.. Bioinformatics.

[63] Swedlow JR, Goldberg IG, Brauner E, Sorger PK (2003). Informatics and quantitative analysis in biological imaging.. Science.

[64] Goldberg IG, Allan C, Burel JM, Creager D, Falconi A (2005). The Open Microscopy Environment (OME) Data Model and XML file: open tools for informatics and quantitative analysis in biological imaging.. Genome Biol.

[65] Kvilekval K, Fedorov D, Obara B, Singh A, Manjunath BS (2009). Bisque: a platform for bioimage analysis and management.. Bioinformatics.

